# Supplementary material for: Identifying priority double-duty actions to tackle the double burden of malnutrition in infants and young children in Peru: Assessment and prioritisation of government actions by national experts
Source: PLoS One. 2024 May 20;19(5):e0303668. doi: 10.1371/journal.pone.0303668 (PMC11104715; doi:10.1371/journal.pone.0303668)
Supplement: S1 Table — (PDF) [file pone.0303668.s001.pdf]

**Policies indicators (double-duty actions)(full description)**	
1. Initiatives to promote and protect exclusive breastfeeding (EBF) in the first 6 month, and beyond<br>Aim: *There is a comprehensive policy implemented by the government to promote exclusive breastfeeding in the first 6 months of life, and beyond.*	MARKETINGFORMULA1: *Effective policies are implemented by the government to restrict exposure and power of promotion of infant formula through <u>broadcast media (TV, radio)</u>*
BREASTFEEDING1: Effective policies are implemented by the government a national policy on BF has been officially adopted/approved by the government, accompanied by an action plan including targets and timeframes to implement and promote the policy.	MARKETINGFORMULA2: *Effective policies are implemented by the government to restrict exposure and power of promotion of infant formula <u>through non-broadcast media</u> (e.g. Internet, social media, food packaging, sponsorship, outdoor advertising including around health clinics)*
BREASTFEEDING2: The international Code of Marketing of Breast Milk Substitutes and the National Code of Marketing of Breast Milk substitutes have been adopted in legislation and been enforced	MARKETINGFORMULA3: *Effective policies are implemented by the government to ensure that infant formula is not commercially promoted <u>in ante-natal and post-natal settings</u>*
BREASTFEEDING3: The national Baby Friendly Hospital Initiative/Ten steps criteria has been adopted and incorporated within the health care system strategies/policy and assessment systems are in place for designating BFHI/Ten steps facilities	4. Maternal nutrition and antenatal care programmes<br>Aim: There is a comprehensive policy implemented by the government to improve the nutritional status of the woman before and during pregnancy. This can reduce the risk of adverse birth outcomes, such as low birthweight and pre-term birth (1).
BREASTFEEDING4: There is paid maternity leave legislation for women	MCH1: Oral supplementation iron and folic acid (IFA supplements) have been established by the government with 30 to 60 mg of elemental iron and 400 µg (0.4 mg) of folic acid for prevent anemia during pregnancy
BREASTFEEDING5:<br>National standards and guidelines for BF promotion and support have been developed and disseminated to all facilities and personnel providing maternity and newborn care.	MCH2: Any nutrition counselling or nutrition counselling (specific content) during pregnancy
BREASTFEEDING6 Community-based breastfeeding outreach and support activities have national coverage	MCH3: Calcium supplementation for pregnant women with low calcium intakes
2.Promotion of appropriate early and complementary feeding in infants	MCH4: Vit A supplementation for pregnant women (low dose for

Aim: *There is a comprehensive policy implemented by the government to improve adequacy of complementary feeding in young infants. The adequacy of complementary feeding refers to timely, adequate, safe and appropriate.*	populations with high prevalence of deficiency)
COMPFEEDING1:<br>- A National policy on infant and young child feeding has been officially adopted<br>- There is a national CF plan of action<br>A national body exists to lead the implementation of the strategy as a coordinated national response	MCH5: Deworming for populations where pregnant women have a 20% or higher prevalence of infection with hookworm or T.trichiura infection AND a 40% or higher prevalence of anaemia
COMPFEEDING2: Counselling for complementary feeding	MCH6: Delayed cord clamping
COMPFEEDING3: Iron supplementation in a population where children of 6-59 months have 20% of higher prevalence of anaemia	5.Pre-School food programmes and policies<br>Aim: *The government ensures that there are healthy food service policies implemented in government funded pre-school settings to ensure that food provision encourages healthy, diverse food consumption that minimise energy dense nutrient poor foods [adapted from* FOOD EPI: 5. FOOD PROVISION and 1. FOOD COMPOSITION]
COMPFEEDING4: Vitamin A supplementation (high dose)	PRESCHOOL1: *Food composition targets/standards have been established <u>for pre-school food service outlets</u> by the government for the content of the nutrients of concern in certain foods or food groups if they are major contributors to population intakes of these nutrients of concern (trans fats, added sugars, salt, saturated fat*) from FOOD EPI-COMP2
COMPFEEDING5: Multiple micronutrient powders (MNP for anaemia) in a population where children of 6-59 months have 20% or higher prevalence of anaemia	PRESCHOOL2: *The government ensures that there are clear, consistent policies (including nutrition standards) implemented in early childhood education services for food service activities (canteens, food at events, fundraising, promotions, vending machines etc.) to provide and promote healthy food choices* from FOOD EPI-PROV1

COMPFEEDING6: Preventive zinc supplementation	PRESCHOOL3 *The Government ensures that there are good support and training systems to help pre-school settings and their caterers meet the healthy food service policies and guidelines* from FOOD EPI-PROV3
3.Regulations on marketing' double duty actions<br>Aim: *There is a comprehensive policy implemented by the government to reduce the impact (exposure and power) of promotion of unhealthy foods to children (<16years) and breast milk substitutes across all media*	
MARKETINGFOOD1: *Effective policies are implemented by the government to restrict exposure and power of promotion of unhealthy foods to children through broadcast media (TV, radio)* NB from Food-EPI	
MARKETINGFOOD2: *Effective policies are implemented by the government to restrict exposure and power of promotion of unhealthy foods to children through non-broadcast media (e.g. Internet, social media, food packaging, sponsorship, outdoor advertising including around schools)* NB from Food-EPI	
MARKETINGFOOD3: *Effective policies are implemented by the government to ensure that unhealthy foods are not commercially promoted to children in settings where children gather (e.g. preschools, sport and cultural events)* NB from Food-EPI	

**Infrastructure support indicators (the enabling environment) (full description)**	
6 LEADERSHIP:<br>The political leadership ensures that there is strong support for the vision, planning, communication, implementation and evaluation of policies and interventions to prevent obesity, stunting and iron deficiency anaemia in young children and reduce nutritional inequalities adapted from FOOD EPI	9 FUNDING AND RESOURCES:<br>Sufficient funding is invested in reducing obesity, stunting and iron deficiency anaemia in young children and their related inequalities adapted from FOOD-EPI

LEAD1: There is strong, visible, political support (at the Head of Government / Cabinet level) for preventing obesity, stunting and iron deficiency anaemia in young children adapted from FOOD-EPI	FUND1: Funding for interventions and policies to reduce obesity, stunting and iron deficiency anaemia in young children as a proportion of total health spending is sufficient to reduce their prevalence and reduce associated inequalities adapted from FOOD-EPI
LEAD2: Clear population intake targets have been established by the government for the nutrients of concern (iron, added sugar, saturated fat and protein) to meet WHO and national recommended dietary intake levels adapted from FOOD-EPI	FUND2: Government funded research is targeted for improving obesity, stunting and iron deficiency anaemia in young children and their related inequalities adapted from FOOD-EPI
LEAD3: Clear, interpretive, evidence-informed food-based dietary guidelines have been established and implemented to promote healthy, diverse diets for young children adapted from FOOD-EPI	FUND3: There is a statutory health promotion agency in place that includes an objective to promote healthy, diverse diets of young children, with a secure funding stream adapted from FOOD-EPI
LEAD4: Government priorities have been established to reduce inequalities or protect vulnerable populations in relation to obesity, stunting and iron deficiency anaemia in young children adapted from FOOD-EPI	10 PLATFORMS FOR INTERACTION: There are coordination platforms and opportunities for synergies across government departments, levels of government, and other sectors (NGOs, private sector, and academia) such that policies and interventions are coherent, efficient and effective in preventing obesity, stunting and iron deficiency anaemia in young children and their related inequalities adapted from FOOD-EPI
7 GOVERNANCE: *Governments have structures in place to ensure transparency and accountability, and encourage broad community participation and inclusion when formulating and implementing policies and interventions to prevent obesity, stunting and iron deficiency anaemia in young children and reduce diet-related inequalities adapted from* FOOD EPI	PLATF1: There are robust coordination mechanisms across departments and levels of government (national and local) to ensure policy coherence, alignment, and integration of policies in obesity, stunting and iron deficiency anaemia in young children policies across governments adapted from FOOD-EPI
GOVER1: There are robust procedures to restrict commercial influences on the development of policies related to preventing obesity, stunting and iron deficiency anaemia in young children adapted from FOOD-EPI	PLATF2: There are formal platforms between government and the commercial food sector to implement healthy food policies to prevent obesity, stunting and iron deficiency anaemia in young children adapted from FOOD-EPI
GOVER2: Policies and procedures are implemented for using evidence in the development of nutrition policies related to obesity, stunting and iron deficiency	PLATF3: There are formal platforms for regular interactions between government and civil society on nutrition policies and other strategies to prevent obesity,

anaemia in young children adapted from FOOD-EPI	stunting and iron deficiency anaemia in young children adapted from FOOD-EPI
GOVER3: The government ensures access to comprehensive nutrition information and key documents (e.g. budget documents, annual performance reviews and health indicators) for the public used from FOOD-EPI	11 HEALTH IN ALL POLICIES: Processes are in place to ensure policy coherence and alignment, and that impacts on obesity, stunting and iron deficiency anaemia in young children are explicitly considered in the development of government policies adapted from FOOD-EPI
8 MONITORING AND INTELLIGENCE: *The government's monitoring and intelligence systems (surveillance, evaluation, research and reporting) are comprehensive and regular enough to assess the status and progress of obesity, stunting and iron deficiency anaemia in young children and their inequalities adapted from* FOOD EPI	HIAP1: There are processes in place to ensure that impacts on obesity, stunting and iron deficiency anaemia in young children in vulnerable populations are considered and prioritised in the development of all government policies relating to food adapted from FOOD-EPI
MONIT1: Monitoring systems, implemented by the government, are in place to regularly monitor food environments (for the five proposed double duty actions above), against codes/guidelines/standards/targets. adapted from FOOD-EPI	HIAP2: There are processes (e.g. health impact assessments) to assess and consider nutrition and health impacts during the development of other non-food policies adapted from FOOD-EPI
MONIT2: There is regular monitoring of young children's nutritional status based on population level intakes against specified intake targets or recommended daily intake levels. adapted from FOOD-EPI	
MONIT3: There is regular monitoring of the status and progress of obesity, stunting and iron deficiency anaemia in young children using anthropometric and biological measurements adapted from FOOD-EPI	
MONIT4: There is sufficient evaluation of major programs and policies to assess effectiveness and contribution to reducing obesity, stunting and iron deficiency anaemia in young children adapted from FOOD-EPI	
MONIT5: Progress towards reducing nutritional inequalities in vulnerable young children's populations are regularly monitored adapted from FOOD-EPI	
