# Supplementary figures and images for: Identifying priority double-duty actions to tackle the double burden of malnutrition in infants and young children in Peru: Assessment and prioritisation of government actions by national experts
Source: PLoS One. 2024 May 20;19(5):e0303668. doi: 10.1371/journal.pone.0303668 (PMC11104715; doi:10.1371/journal.pone.0303668)

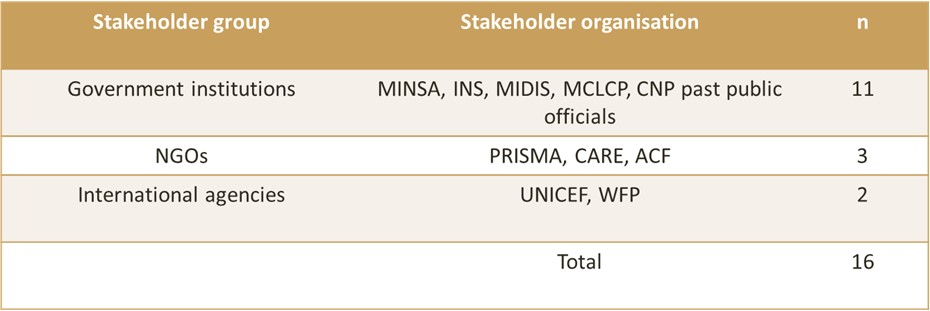

Supplement: S3 Table — (TIFF) [file pone.0303668.s003.tiff]

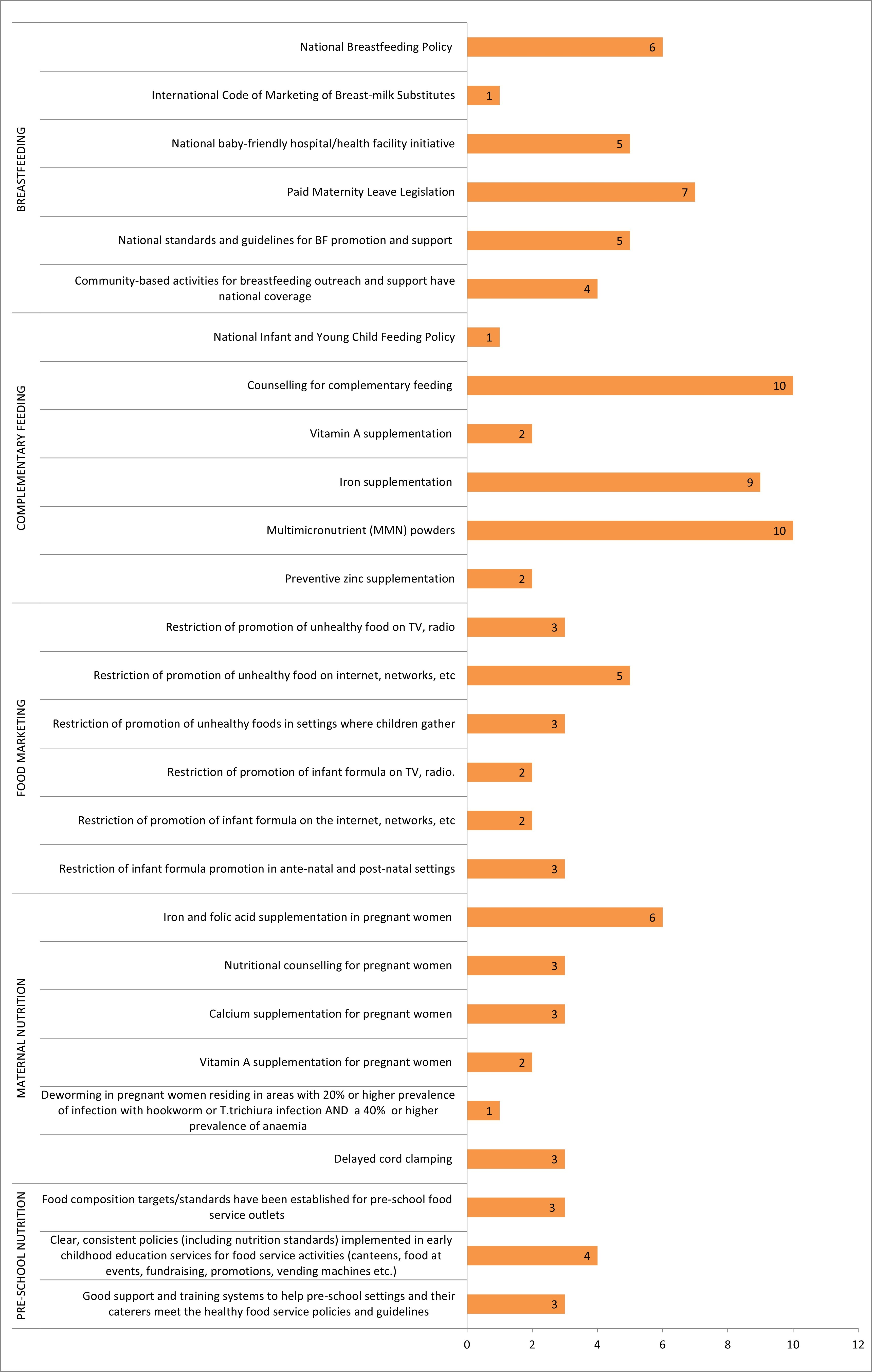

Supplement: S1 Fig — (TIFF) [file pone.0303668.s007.tiff]

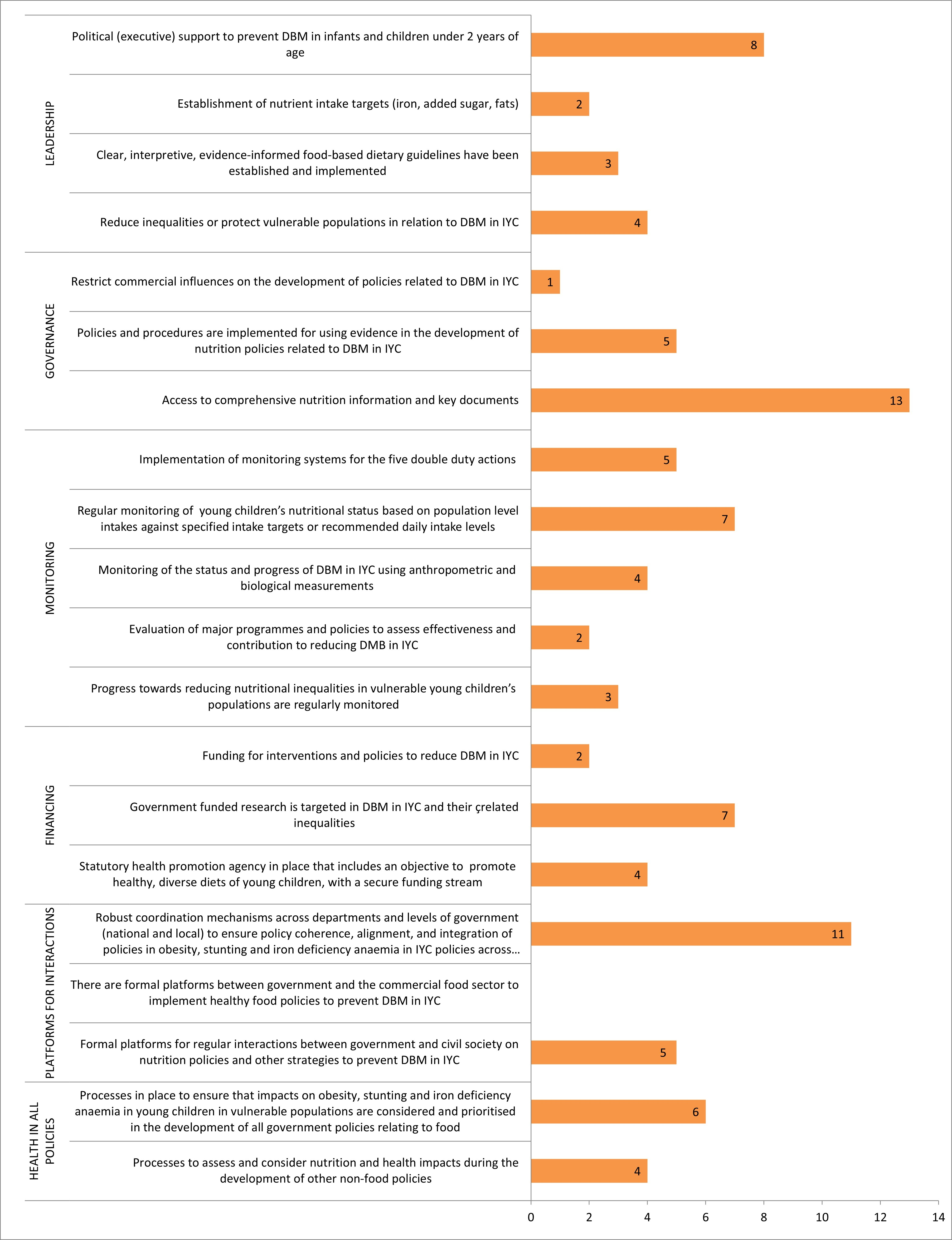

Supplement: S2 Fig — (TIFF) [file pone.0303668.s008.tiff]
