# Supplementary material for: Identifying priority double-duty actions to tackle the double burden of malnutrition in infants and young children in Peru: Assessment and prioritisation of government actions by national experts
Source: PLoS One. 2024 May 20;19(5):e0303668. doi: 10.1371/journal.pone.0303668 (PMC11104715; doi:10.1371/journal.pone.0303668)
Supplement: S4 Table — (DOCX) [file pone.0303668.s004.docx]

| Nombre | Descripción | Archivos | Referencias |
| --- | --- | --- | --- |
| 1. Policy domains |  | 0 | 0 |
| 1.1. Breastfeeding |  | 0 | 0 |
| a. Barriers |  | 0 | 0 |
| Beneficiaries follow-up |  | 0 | 0 |
| Beneficiaries of the policy |  | 0 | 0 |
| Health, economic and social context |  | 4 | 7 |
| Human resources |  | 0 | 0 |
| Implementers |  | 1 | 1 |
| Policy makers |  | 2 | 2 |
| Policy monitoring and evaluation |  | 11 | 16 |
| Political constraints |  | 1 | 2 |
| Supervision, control and sanctions for regulatory compliance |  | 10 | 15 |
| Supplies and equipment |  | 0 | 0 |
| b. Facilitators |  | 0 | 0 |
| Beneficiaries of the policy |  | 0 | 0 |
| Beneficiaries follow-up |  | 0 | 0 |
| Health, economic and social context |  | 0 | 0 |
| Human resources |  | 0 | 0 |
| Implementers |  | 0 | 0 |
| Policy makers |  | 0 | 0 |
| Policy monitoring and evaluation |  | 3 | 3 |
| Political opportunities |  | 0 | 0 |
| Supervision, control and sanctions for regulatory compliance |  | 1 | 1 |
| Supplies and equipment |  | 0 | 0 |
| c. Recommendations |  | 2 | 3 |
| 1.2. Complementary feeding |  | 0 | 0 |
| a. Barriers |  | 0 | 0 |
| Beneficiaries follow-up |  | 0 | 0 |
| Beneficiaries of the policy |  | 1 | 1 |
| Health, economic and social context |  | 2 | 2 |
| Human resources |  | 0 | 0 |
| Implementers |  | 0 | 0 |
| Policy makers |  | 0 | 0 |
| Policy monitoring and evaluation |  | 7 | 15 |
| political constraints |  | 1 | 1 |
| Supervision, control and sanctions for regulatory compliance |  | 6 | 10 |
| Supplies and equipment |  | 2 | 4 |
| b. Facilitators |  | 0 | 0 |
| Beneficiaries of the policy |  | 0 | 0 |
| Health, economic and social context |  | 0 | 0 |
| Human resources |  | 0 | 0 |
| Implementers |  | 0 | 0 |
| Policy makers |  | 0 | 0 |
| Policy monitoring and evaluation |  | 0 | 0 |
| Political opportunities |  | 0 | 0 |
| Supervision, control and sanctions for regulatory compliance |  | 0 | 0 |
| Supplies and equipment |  | 0 | 0 |
| c. Recommendations |  | 4 | 6 |
| 1.3. Food & formula marketing |  | 0 | 0 |
| a. Barriers |  | 0 | 0 |
| Beneficiaries follow-up |  | 0 | 0 |
| Beneficiaries of the policy |  | 3 | 3 |
| Health, economic and social context |  | 2 | 2 |
| Human resources |  | 0 | 0 |
| Implementers |  | 2 | 2 |
| Policy makers |  | 0 | 0 |
| Policy monitoring and evaluation |  | 8 | 13 |
| political constraints |  | 0 | 0 |
| Supervision, control and sanctions for regulatory compliance |  | 7 | 11 |
| Supplies and equipment |  | 2 | 2 |
| b. Facilitators |  | 0 | 0 |
| Beneficiaries of the policy |  | 0 | 0 |
| Health, economic and social context |  | 0 | 0 |
| Human resources |  | 0 | 0 |
| Implementers |  | 0 | 0 |
| Policy makers |  | 0 | 0 |
| Policy monitoring and evaluation |  | 0 | 0 |
| Political opportunities |  | 0 | 0 |
| Supervision, control and sanctions for regulatory compliance |  | 1 | 1 |
| Supplies and equipment |  | 0 | 0 |
| c. Recommendations |  | 4 | 4 |
| 1.4. Maternal nutrition indicator |  | 0 | 0 |
| a. Barriers |  | 0 | 0 |
| Beneficiaries follow-up |  | 0 | 0 |
| Beneficiaries of the policy |  | 0 | 0 |
| Health, economic and social context |  | 1 | 1 |
| Human resources |  | 0 | 0 |
| Implementers |  | 1 | 1 |
| Policy makers |  | 0 | 0 |
| Policy monitoring and evaluation |  | 5 | 6 |
| political constraints |  | 0 | 0 |
| Supervision, control and sanctions for regulatory compliance |  | 7 | 10 |
| Supplies and equipment |  | 1 | 1 |
| b. Facilitators |  | 0 | 0 |
| Beneficiaries of the policy |  | 0 | 0 |
| Health, economic and social context |  | 0 | 0 |
| Human resources |  | 0 | 0 |
| Implementers |  | 0 | 0 |
| Policy makers |  | 0 | 0 |
| Policy monitoring and evaluation |  | 1 | 1 |
| Political opportunities |  | 0 | 0 |
| Supervision, control and sanctions for regulatory compliance |  | 1 | 2 |
| Supplies and equipment |  | 0 | 0 |
| c. Recommendations |  | 1 | 1 |
| 1.5. Preschool indicator |  | 0 | 0 |
| a. Barriers |  | 0 | 0 |
| Beneficiaries follow-up |  | 0 | 0 |
| Beneficiaries of the policy |  | 1 | 1 |
| Health, economic and social context |  | 2 | 2 |
| Human resources |  | 0 | 0 |
| Implementers |  | 1 | 1 |
| Policy makers |  | 0 | 0 |
| Policy monitoring and evaluation |  | 5 | 6 |
| political constraints |  | 0 | 0 |
| Supervision, control and sanctions for regulatory compliance |  | 4 | 5 |
| Supplies and equipment |  | 0 | 0 |
| b. Facilitators |  | 0 | 0 |
| Beneficiaries of the policy |  | 0 | 0 |
| Health, economic and social context |  | 0 | 0 |
| Human resources |  | 0 | 0 |
| Implementers |  | 0 | 0 |
| Policy makers |  | 0 | 0 |
| Policy monitoring and evaluation |  | 0 | 0 |
| Political opportunities |  | 0 | 0 |
| Supervision, control and sanctions for regulatory compliance |  | 0 | 0 |
| Supplies and equipment |  | 0 | 0 |
| c. Recommendations |  | 1 | 1 |
| 2. Infrastructure support |  | 0 | 0 |
| 2.1. Leadership indicator |  | 0 | 0 |
| a. Barriers |  | 0 | 0 |
| Beneficiaries follow-up |  | 0 | 0 |
| Beneficiaries of the policy |  | 0 | 0 |
| Health, economic, political and social context |  | 3 | 3 |
| Human resources |  | 0 | 0 |
| Implementers |  | 3 | 4 |
| Policy makers |  | 0 | 0 |
| Policy monitoring and evaluation |  | 5 | 6 |
| Political constrains |  | 2 | 2 |
| Supervision, control and sanctions for regulatory compliance |  | 2 | 2 |
| Supplies and equipment |  | 0 | 0 |
| b. Facilitators |  | 0 | 0 |
| Beneficiaries of the policy |  | 0 | 0 |
| Health, economic and social context |  | 0 | 0 |
| Human resources |  | 0 | 0 |
| Implementers |  | 0 | 0 |
| Policy makers |  | 0 | 0 |
| Policy monitoring and evaluation |  | 1 | 1 |
| Political opportunities |  | 0 | 0 |
| Supervision, control and sanctions for regulatory compliance |  | 0 | 0 |
| Supplies and equipment |  | 0 | 0 |
| c. Recommendations |  | 5 | 10 |
| 2.2. Governance indicator |  | 0 | 0 |
| a. Barriers |  | 0 | 0 |
| Beneficiaries follow-up |  | 0 | 0 |
| Beneficiaries of the policy |  | 0 | 0 |
| Health, economic and social context |  | 1 | 1 |
| Human resources |  | 0 | 0 |
| Implementers |  | 3 | 3 |
| Policy makers |  | 0 | 0 |
| Policy monitoring and evaluation |  | 7 | 7 |
| Political constrains |  | 0 | 0 |
| Supervision, control and sanctions for regulatory compliance |  | 6 | 7 |
| Supplies and equipment |  | 0 | 0 |
| b. Facilitators |  | 0 | 0 |
| Beneficiaries of the policy |  | 0 | 0 |
| Health, economic and social context |  | 0 | 0 |
| Human resources |  | 0 | 0 |
| Implementers |  | 1 | 1 |
| Policy makers |  | 0 | 0 |
| Policy monitoring and evaluation |  | 0 | 0 |
| Political opportunities |  | 2 | 2 |
| Supervision, control and sanctions for regulatory compliance |  | 0 | 0 |
| Supplies and equipment |  | 0 | 0 |
| c. Recommendations |  | 3 | 5 |
| 2.3. Monitoring indicator |  | 0 | 0 |
| a. Barriers |  | 0 | 0 |
| Beneficiaries follow-up |  | 0 | 0 |
| Beneficiaries of the policy |  | 0 | 0 |
| Health, economic and social context |  | 1 | 1 |
| Human resources |  | 0 | 0 |
| Implementers |  | 0 | 0 |
| Policy makers |  | 0 | 0 |
| Policy monitoring and evaluation |  | 2 | 2 |
| Political constrains |  | 0 | 0 |
| Supervision, control and sanctions for regulatory compliance |  | 2 | 2 |
| Supplies and equipment |  | 0 | 0 |
| b. Facilitators |  | 0 | 0 |
| Beneficiaries of the policy |  | 0 | 0 |
| Health, economic and social context |  | 0 | 0 |
| Human resources |  | 0 | 0 |
| Implementers |  | 0 | 0 |
| Policy makers |  | 0 | 0 |
| Policy monitoring and evaluation |  | 2 | 2 |
| Political opportunities |  | 1 | 1 |
| Supervision, control and sanctions for regulatory compliance |  | 1 | 3 |
| Supplies and equipment |  | 0 | 0 |
| c. Recommendations |  | 2 | 3 |
| 2.4. Financing indicator |  | 0 | 0 |
| a. Barriers |  | 0 | 0 |
| Beneficiaries follow-up |  | 0 | 0 |
| Beneficiaries of the policy |  | 0 | 0 |
| Health, economic and social context |  | 3 | 4 |
| Human resources |  | 0 | 0 |
| Implementers |  | 1 | 1 |
| Policy makers |  | 0 | 0 |
| Policy monitoring and evaluation |  | 3 | 5 |
| Political constrains |  | 2 | 2 |
| Supervision, control and sanctions for regulatory compliance |  | 2 | 2 |
| Supplies and equipment |  | 1 | 1 |
| b. Facilitators |  | 0 | 0 |
| Beneficiaries of the policy |  | 0 | 0 |
| Health, economic and social context |  | 0 | 0 |
| Human resources |  | 0 | 0 |
| Implementers |  | 0 | 0 |
| Policy makers |  | 0 | 0 |
| Policy monitoring and evaluation |  | 0 | 0 |
| Political opportunities |  | 0 | 0 |
| Supervision, control and sanctions for regulatory compliance |  | 1 | 1 |
| Supplies and equipment |  | 1 | 1 |
| c. Recommendations |  | 2 | 2 |
| 2.5. Platforms for interaction |  | 0 | 0 |
| a. Barriers |  | 0 | 0 |
| Beneficiaries follow-up |  | 0 | 0 |
| Beneficiaries of the policy |  | 0 | 0 |
| Health, economic and social context |  | 0 | 0 |
| Human resources |  | 0 | 0 |
| Implementers |  | 0 | 0 |
| Policy makers |  | 0 | 0 |
| Policy monitoring and evaluation |  | 5 | 6 |
| Political constrains |  | 1 | 2 |
| Supervision, control and sanctions for regulatory compliance |  | 2 | 3 |
| Supplies and equipment |  | 0 | 0 |
| b. Facilitators |  | 0 | 0 |
| Beneficiaries of the policy |  | 0 | 0 |
| Health, economic and social context |  | 0 | 0 |
| Human resources |  | 0 | 0 |
| Implementers |  | 0 | 0 |
| Policy makers |  | 0 | 0 |
| Policy monitoring and evaluation |  | 0 | 0 |
| Political opportunities |  | 0 | 0 |
| Supervision, control and sanctions for regulatory compliance |  | 0 | 0 |
| Supplies and equipment |  | 0 | 0 |
| c. Recommendations |  | 2 | 2 |
| 2.6. Health in all policies |  | 0 | 0 |
| a. Barriers |  | 0 | 0 |
| Beneficiaries follow-up |  | 0 | 0 |
| Beneficiaries of the policy |  | 0 | 0 |
| Health, economic and social context |  | 0 | 0 |
| Human resources |  | 0 | 0 |
| Implementers |  | 0 | 0 |
| Policy makers |  | 0 | 0 |
| Policy monitoring and evaluation |  | 2 | 2 |
| Political constrains |  | 0 | 0 |
| Supervision, control and sanctions for regulatory compliance |  | 2 | 2 |
| Supplies and equipment |  | 0 | 0 |
| b. Facilitators |  | 0 | 0 |
| Beneficiaries of the policy |  | 0 | 0 |
| Health, economic and social context |  | 0 | 0 |
| Human resources |  | 0 | 0 |
| Implementers |  | 0 | 0 |
| Policy makers |  | 0 | 0 |
| Policy monitoring and evaluation |  | 0 | 0 |
| Political opportunities |  | 1 | 1 |
| Supervision, control and sanctions for regulatory compliance |  | 0 | 0 |
| Supplies and equipment |  | 0 | 0 |
| c. Recommendations |  | 1 | 1 |
