# Supplementary material for: Identifying priority double-duty actions to tackle the double burden of malnutrition in infants and young children in Peru: Assessment and prioritisation of government actions by national experts
Source: PLoS One. 2024 May 20;19(5):e0303668. doi: 10.1371/journal.pone.0303668 (PMC11104715; doi:10.1371/journal.pone.0303668)
Supplement: S5 Table — (DOCX) [file pone.0303668.s005.docx]

| **Double-Duty action** | **Reasons for prioritisation** | | **Representatives Excerpts** |
| --- | --- | --- | --- |
| A national policy on BF has been officially adopted/approved by the government, accompanied by an action plan including targets and timeframes to implement and promote the policy | Easy implementation  Relevance | The fact that this policy already has a track record and is not starting from scratch  The experts highlighted that the breastfeeding committee of the Ministry of Health has identified the need to work on a breastfeeding promotion law to give more support to breastfeeding because the existing regulations are often weak, especially on the issue of breastmilk substitutes.  The importance of breastfeeding | *“There are already actions in place on the issue of breastfeeding, so I believe that this official adoption of a national exclusive breastfeeding policy accompanied by an action plan is feasible.” (National representative, government sector)*  *“There is a breastfeeding issue, in the Ministry of Health there is a breastfeeding committee, and this committee feels very limited because they believe that they should have a law to promote breastfeeding and at the same time avoid the whole issue of milk substitutes that are entering with some force at the first level of care and it seems that the existing regulations are usually weak, so there is an issue of working on something more powerful”. (National representative, government sector)*  *“There is sufficient evidence to support that it (breastfeeding) is a practice that should be implemented to contribute to reduce the problems of the double burden of malnutrition.” (National representative, government sector* |
| The national Baby Friendly Hospital Initiative/Ten steps criteria has been adopted and incorporated within the health care system strategies/policy and assessment systems are in place for designating BFHI/Ten steps facilities | Established regulation  Available budget  Relevance | The regulation has already been implemented years ago. Experts also pointed that it needed a hard and constantly work  There is a budget earmarked for this action  The impact has been verified in the accredited hospitals | *“**Years ago, we had many mother and child friendly hospitals, but then all of them ceased to be so, and again MINSA, before the pandemic, was taking up this aspect again and some hospitals began to be accredited, however, it is hard work, it is a job in which there has to be a lot of commitment.” (National representative, government sector)*  *“For this action there is a budget, as we have been working for many years in the MINSA management agreements and there is an indicator that has incorporated the issue of certification of health facilities and the mother and child-friendly.” (National representative, government sector)*  *“In hospitals where pre-pandemic assessments were conducted, there were changes in three key indicators: umbilical cord cutting, late clamping, skin-to-skin contact and breastfeeding in the first hour.”(National representative, government sector)* |
| Paid maternity leave legislation | Easy implementation  Relevance | There are initiatives such as maternity or paternity leave  It is worth noting that the experts highlighted that there is a gap with regard to women working in the informal sector as they do not receive this benefit as well as women working in the public sector under outsourced services.  That it will have an impact on breastfeeding | *“As far as I understand it, the mother is given breastfeeding leave, she can enter an hour later or leave an hour earlier without affecting her remuneration, now we are talking about a legislative proposal to remunerate the person when she is in the maternity period, I believe that the impact would be very high.”(National representative, non-governmental organization)*  *“While it is true that the country has regulated the issue of maternity leave for all women who are linked to any entity, both public and private, they enjoy this leave and have a remuneration, but there is a big gap in the issue of informality, as women working informally who can enter in this state are not benefited, even public sector workers who work under the modality of outsourced services do not receive this benefit, I think it is a pending issue that we have to work with the Ministry of Labour.” (National representative, government sector)* |
| The national standards and guidelines for BF promotion and support | Easy implementation  Established regulation | Their technical procedures are very easily achieved  *It should be noted that experts point out that there is a slow process of implementation of the regulations, which could hinder their implementation.*  They are supported by the previous existence of infant and young child feeding guidelines that include breastfeeding | *“I believe that it has a high impact and high feasibility, guidelines and standards are being worked on, and even feeding guidelines for children under two years of age have been published, which also include breastfeeding issues, so I believe that we are making progress in terms of regulations.” (National representative, non-governmental organization)*  *As these types of regulations are implemented at the first level of care, it is necessary to consider that the regulations issued by the Ministry take more or less 18 to 24 months to go through the whole process of reverse scaling, that is, the regulation that is generated in the Ministry, from there it has to go to the regional directorates, from there to the micro networks, networks, establishments, and when the personnel of the first level of care find out that there is a new regulation, they are trained and modify their practice, it is a slow process. ” (National representative, non-governmental organization)*  *“I see that it has a high impact and a very high feasibility, i.e., the generation of technical standards or procedures is very high, it is very easy to get, materials are generated, people take it up, so there is a very high feasibility.” (National representative, international agency)* |
| Community-based activities for breastfeeding outreach and support | Established regulation  Relevance | Regulations on breastfeeding are in place  The experts highlight the high impact of community activities, but also explain the need for health personnel to understand the essence of community work, which would require sensitisation and training of health personnel.  Community work like that, has a lot of impact | *“There are already established norms for this, and the impact is quite high. For example, when I was in the establishment, I followed up on children who had low birth weight or were premature, and the community agents were the ones who visited the mothers, I could say that all my children recovered despite their conditions at birth, they recovered, after two or three months they were the right size and weight. As for feasibility, I would put it high, based on what I have experienced as a health worker, it depends on the motivation given to the staff.” (National representative, government sector)*  *“A community activity basically shows the interlocutor as a peer and not as an expert, so if I am going to do a community activity and I am going to put as a model a woman from the community who breastfeeds her child and who can be showing positive effects of this, this possibility of an interaction between parents in positive deviance, finding a role model who is like you, understanding community work in this way, has a lot of impact.... Unfortunately, in Peru, especially in public institutions, we have not yet finished understanding what community processes are like, so it doesn't go beyond a banner, a perifoneo and they don't get fully involved in the community dynamics, the famous extra-mural work doesn't end up doing more than extension activities of the health service, but without changing the community logic, those processes that we understand as slow, reflexive, back and forth processes, and that is difficult to understand...” (National representative, non-governmental organization)*  *“That impact is very high, because if we are talking about implementation of community activities, I understand that a community activity basically shows the interlocutor as a peer and not an expert, so if I am going to do a community activity and I am going to put as a model a lady from the community who breastfed her child and who may be showing positive effects of it, that possibility of a peer interaction is the positive relationship, finding a role model who is like you, I understanding community work like that, has a lot of impact.” (National representative, government sector)* |
| Counselling for complementary feeding | Available budget  Relevance | There is a budget at national, regional, and local level  It is cost-effective, and they consider that a complementary feeding counselling has a high impact.  It should be noted that the experts pointed out that there are difficulties inherent to the health system that hinder the optimal implementation of counselling, in terms of time and available human resources, the demands of meeting targets associated with service coverage rather than oriented to impact. | *“Feasibility is high, moreover, we are emphasizing quite a lot this intervention, if there is budget at national level, at different levels of national, regional and local government, in terms of impact also high, because we have ensured the issue of counselling.” (National representative, government sector)*  *“That does have high impact and it is also highly feasibility because it is cost-effective.” (National representative, government sector)*  *"It is very difficult to do nutritional counselling in Peru with the conditions that would be required to achieve this impact, if the counselling is intended to be done in the facility as part of a service, the amount of activities that health personnel do at the first level of care leaves very little time to do nutritional counselling that generates impact. ... the steps for counselling are not taken is difficult to implement and this is not only due to the attitudes and capacities of the staff but also to the system, a health system in which productivity is sought at all costs, to achieve high coverage that does not give time to processes that are really slow if you want to find impacts". (National representative, non-governmental organization)* |
| Restriction of promotion of unhealthy foods in settings where children gather | Established regulation  Easy implementation | There are already regulations in place  Easy implementation and control | *“There are healthy school kiosks, the regulations are there, with good supervision, the impact would be high and the viability would also be high because to some extent there are all the regulations and it is the schools themselves that must enforce them.” (National representative, government sector)*  *“The viability is high because they are concrete events, so you know where, when and who and you can generate a list of events to promote those moments and these foods can be restricted, because when you have already defined events you can implement a mechanism of attention and distribution, so it is more viable.” (National representative, non-governmental organization)* |
| Iron and folic acid supplementation in pregnant women | Established regulation  Available budget  Relevance | It is an intervention that is already being implemented  It has a budget allocation through the Articulated Nutritional Programme and the Maternal and Perinatal Health Programme.  it is an effective intervention. Furthermore, depend on the provision of supplements to mothers, adherence to supplementation, follow-up, and sensitisation of health personnel  Experts pointed out the need to have better control of adherence to supplementation to achieve a positive impact during pregnancy | *"The* *feasibility is high, as long as it is tied to the prenatal maternal health programme and also in the Articulated Nutritional Programme, it means that there is budget and, high impact, because it is one of the effective interventions for the issue of anaemia." (National representative, government sector)*  *"Well, this is already happening and here I would say high impact and high feasibility, because it is already happening, the country has iron and folic acid supplementation free of charge for pregnant women." (National representative, government sector)*  *"The expected impact is very high, the feasibility is also high, but it depends on the adherence to iron supplementation in pregnant women, in the health establishment there is provision of supplementation, but consumption is still low among pregnant women and in this context of the COVID-19 pandemic the provision and adherence to supplementation is being affected." (National representative, government sector)*  *"The contingency is whether we are looking at it as a service provision, but once again, adherence to the supplement can reduce the impact in a very impactful way. In theory, implementing iron and folic acid supplementation during pregnancy has a very high impact, but it is not enough for the establishment or however the supplement reaches the pregnant woman, the important thing is that she consumes it, and unfortunately we still do not have much control over this." (National representative, non-governmental organization)* |
| Nutritional counselling for pregnant women | Relevance  Established guidelines | It has high impact because the nutrition counselling sessions cover a range of issues  There are guidelines for implementation  It is worth noting that the experts pointed out different aspects that need to be improved in order to facilitate the impact of counselling on pregnant women, such as training of health personnel, key messages, staff and time available, follow-up, structure, appropriate space. | *“Here the impact is very high, because I can teach during counselling the importance of breastfeeding during the first hour after birth, clamping, I can talk to them about nutrition in pregnancy, about breastfeeding.” (National representative, government sector)*  *“It is high impact, and it is also high in terms of feasibility, because it is already known, there are even guidelines for good monitoring and promotion, and exit interviews with the pregnant woman could be implemented, which would be the best way to know about feasibility.” (National representative, government sector)*  *"If the counselling is done with follow-up, but if it is only going to be part of giving information, then the staff is not trained in the methodological steps of counselling, because counselling is not only giving information, it is a whole methodological and thematic process. (National representative, government sector)*  *"There is a good disposition on the part of the mothers but I think that the messages need to be worked on, that is to say, they tell them anything, sometimes they tell them to stop eating because you are going to gain weight and your child is going to grow a lot, so this is terrible counselling when this learning takes place." (National representative, government sector)*  *"Basically you need to take into account the conditions such as the time available for the health personnel to do the counselling, structure and appropriate space everything." (National representative, non-governmental organization)* |
| Delayed cord clamping | Relevance  Easy implementation | It is a cost-effectiveness intervention, and it has the many advantages for the newborn.  It has high feasibility, but the success depends on motivation and staff confidence. | *“This is a jewel that we are not appreciating and that is the most cost-effective thing there is from birth, the expected impact would be very high.” (National representative, government sector)*  *“The feasibility will depend on the extent to which we as health personnel can inform the pregnant woman herself and as civil society, we can also inform the mothers about the advantages of these three activities during, as they say, the golden hour of the newborn, so from that point of view i would say high feasibility.” (National representative, government sector)*  *“That does have a very high impact, I don't even think about it. the feasibility of doing it also depends on the motivation of the staff, you must trust them." (National representative, government sector)* |
| The government ensuring that there are clear, consistent policies (including nutrition standards) implemented in early childhood education services for food service activities to provide and promote healthy food choices (note: this the only action in early implementation stage), | Relevance  Easy implementation | It has a high impact due to the focus on providing good nutrition during the first two years of life  The fact that this action involves institutions could facilitate the implementation of this action.  The experts also pointed out that there would be some limitations to its implementation as it is necessary to consider the need for infrastructure and budget to take Cuna Mas Programme to a national scale. | *“If we are talking about the programme Cuna Más, obviously the expected impact is high because the first two years are very important in terms of nutrition.” (National representative, government sector)*  *“Feasibility can be high, as the action of providing and promoting healthy food in an already institutionalized facility requires incorporation into the policy guidelines and operating rules of the facility. This should work well, and feasibility is only limited by very specific infractions or lack of awareness.” (National representative, non-governmental organization)*  *“We are talking about cycle 1^1^ and Cuna Más^2^ in this segment I cannot pack it and bring the food, it has to be prepared there, that makes it more complicated, that is why I consider it medium viability, especially the implementation I see as complicated, it would have to include infrastructure, Cuna Más currently does it but it does it on a small scale, taking it to a large scale is a challenge, a very long gap to jump over.” (National representative, international organization)*  *^1^Regular Basic Education of the Peruvian Education System includes the initial non-school level for children from 0 to 2 years of age.*  *^2^The National Programme Cuna Más aims to improve the development of children under 36 months of age in localities in poverty and extreme poverty.* |
