# Supplementary material for: Identifying priority double-duty actions to tackle the double burden of malnutrition in infants and young children in Peru: Assessment and prioritisation of government actions by national experts
Source: PLoS One. 2024 May 20;19(5):e0303668. doi: 10.1371/journal.pone.0303668 (PMC11104715; doi:10.1371/journal.pone.0303668)
Supplement: S6 Table — (DOCX) [file pone.0303668.s006.docx]

| **Infrastructure support action** | **Reasons for prioritisation** | | **Representatives Excerpts** |
| --- | --- | --- | --- |
| Political (executive) support to prevent DBM in infants and young children under 2 years of age | Established regulation  Relevance | The fact that several related policies have been implemented recently, which reinforces the feasibility of this action.    In addition, the experts mentioned that the various political organisations have expressed their concern about these issues, which gives support for these policies. | "*The feasibility I would put it as high because now we have the policy "Peru, healthy country” and this gives it strength, this is telling us that political support is present.*" *(National representative, government sector)*  "*In the case of the law 30021, so far there is political support, we have achieved first that the law is put in place, that the law comes out, it was not easy, private enterprise made us kick quite a lot for it to come out...we will continue to need a lot of political support so the impact is high and the feasibility is also high*." *(National representative, government sector)*  "*The expected impact is high, the feasibility could be high depending on the new congress, the good thing that has been seen is that the political parties have incorporated the issue of anaemia and the issue of the fight against hunger, I think the majority have done so, I think it is an issue on which there is more consensus*." *(National representative, government sector)* |
| Clear, interpretive, evidence-informed food-based dietary guidelines have been established and implemented. | Established regulation  Easy implementation | The experts agreed that the feasibility is high because the guidelines are already published and accessible to all.  The experts also noted that the feasibility and expected impact is high or very high because these are issues that have been worked on and the guidelines are already in place.  However, the experts also pointed out that there are difficulties in monitoring and support to ensure that this and other guidelines are used by health workers during counselling. | "*The recently published dietary guidelines are very similar to the ones we were already working on, there is only one or two variations, for example, the issue of salt, sugar and water. I would give them a high impact because they are issues that have already been worked on, and in terms of feasibility they are also high*. " *(National representative, government sector)*  "*The expected impact is high, thinking about the health providers and other social protection platforms that should use this, the feasibility is also very high, in fact, the guidelines are already published, now they have to be used*." *(National representative, international agency)*  "*There the impact and feasibility should be high, what happens is that in Peru’s experience, we have so many guidelines that are not implemented, it is very difficult for the sector to implement all these instruments because there is no follow-up and support structure. There is no permanent monitoring and support structure to ensure that they are implemented.* " *(National representative, international agency)* |
| Reduce inequalities or protect vulnerable populations in relation to obesity, stunting and iron deficiency anaemia in young children | Similar actions established | the experts pointed out that among the reasons for rating it as high impact and feasible is the fact that there are priorities and actions already being taken as part of the fight against anaemia and chronic child undernutrition. | "*Very high impact and feasibility, because it is about establishing priorities, hand in hand with the guarantor of rights which is the government, they appoint a commission and what the commission does is to establish priorities and propose actions for that, a large part is being done with the issue of chronic child malnutrition and anaemia, In fact, the data in the Demographic Health Survey has already come out, focused on the fulfilment of the Budgetary Programmes by Results, so for next year I don't see anything that would prevent it, rather it seems to me that it will continue*." *(National representative, international agency)* |
| Policies and procedures are implemented for using evidence in the development of nutrition policies related to obesity, stunting and iron deficiency anaemia in young children | Similar actions established | Experts agree that the impact and feasibility is high since evidence-based policy design is currently being prioritised and therefore would not require additional effort. | "*I think the expected impact is very high and feasibility between high and very high because these issues are still being prioritised, and there are just proposals from civil society, the national agreement to address all these issues*." *(National representative, government sector)*  "*Very high impact and high feasibility, in the sense that elaborating a policy is different from implementing it, here we are talking about design and Peru normally has a very strong trend of designing its policies, based on evidence, the apparatus itself now behaves like this, so I don't see it as anything that requires an additional effort to comply with what is already in place, rather it is more like putting more enthusiasm into it*." *(National representative, international agency)* |
| Implementation of monitoring systems for the five double duty actions | Relevance  Easy implementation | Experts mentioned that it would have a high or very high impact because it would provide the necessary tools in a single portal to be able to evaluate and see whether a policy is working well or not, to subsequently take the necessary actions and correct errors.  the experts indicated that the feasibility is high due to the different monitoring systems that currently exist where technology helps a lot.  Experts also pointed out that there is a need to ensure that all information is complemented in a single information system, as actions are carried out in different ministries. In addition, they pointed out that it is necessary to designate the institution responsible for the development of the system. | "*The impact would be high because it would provide decision makers with tools to reinforce, strengthen, formulate, correct or ally processes in the implementation of actions, the feasibility I also see it high because it is not complicated, that is now we already see that the Ministry of Development and Social Inclusion, the Ministry of Health among other sectors have public mechanisms for monitoring progress and indicators, technology helps for handling large databases, it is becoming easier and easier this implementation of monitoring systems*." *(National representative, non-governmental organization)*  "*The expected impact is high, and feasibility is also high, because if we already have sources of information, it would be necessary to articulate it and present it in a single portal, all this monitoring information could be grouped together, but it depends a lot on the interest and willingness of the authorities to do so.*" *(National representative, government sector)*.  "*The impact is high, obviously, the feasibility would depend on who does it, we are seeing that all the actions take place in different ministries, we would have to see who does it and that is complex.*"*(National representative, government sector)* |
| Monitoring of the status and progress of obesity, stunting and iron deficiency anaemia in young children using anthropometric and biological measurements | Easy implementation  Similar actions established  Available budget | The experts note that the feasibility is high due to the simplicity of incorporating anthropometric and biochemical data into national surveys for analysis and that the resources to do so are available.  The experts point out that this is being done adequately by the INEI (National Institute of Statistics and Informatics) and the CENAN (National Food and Nutrition Centre).  On the other hand, the experts pointed out that one of the difficulties is the analysis and elaboration of the reports in a timely manner. | "*Very high impact and feasibility because if we are going to see between anthropometric and biochemical measures in both cases, it is simple to incorporate into national surveys, there is already an apparatus set up is to incorporate a couple of indicators, especially the analysis, because there you are taking anthropometry, in fact sub-samples are taken for chronic non-communicable diseases, it is a matter of fine-tuning some instruments and riding on a horse that is already underway, there is not much difficulty*." *(National representative, non-governmental organization)*  "*The INEI and CENAN collect them all the time, in other words the data exists, the issue is that someone is in charge of analysing and publishing them, that generates time lags, so I feel that here the collection already exists, the money exists, the technique exists, the expected impact should be high and the feasibility equally high.*" *(National representative, international agency)*. |
| Progress towards reducing nutritional inequalities in vulnerable young children's populations are regularly monitored | Easy implementation | the experts agree that the feasibility is high because of the access to information provided thanks to INEI (National Institute of Statistics and Informatics) and the CENAN (National Food and Nutrition Centre) , which show ordered and classified information about on the level of poverty, area of residence and participation in social programmes that helps with monitoring.  The experts highlighted that more analysis of data and reporting on results at regional and local level would be needed  It is worth noting that the experts pointed out that one of the things that would contribute to the implementation is to further analyse the existing data and on the other hand that the respective governmental institution should take the decision to do the monitoring. | "*It would have a high impact and feasibility, about that we also have data, for example, in the ENDES they talk about children who are in the first quintile or in the fifth quintile.... So, I think that just as in the previous one, the information is there, we need to see what is happening, to look further, like why such and such region reduced and why the other one did not, but we need to analyse it more in depth and exploit it*." *(National representative, government sector)*  "*That impact should be high and the feasibility very high, in INEI and CENAN that could be done very easily, they could produce it every year as part of a regular guide, already assembled the code in the software they use, then the base and everything would be ready, then that should be very high in its feasibility as it is a very low cos*t." *(National representative, international agency)*  "*We are referring to the population that the Ministry of Development and Social Inclusion sees, which is very small. Well, that could suddenly have a high impact, feasibility as it is a small and more controlled population, it could be HIGH, if they decide to do it, because that is where the issue of monitoring comes in again*.” *(National representative, government sector)* |
| Funding for interventions and policies to reduce obesity, stunting and iron deficiency anaemia in young children | Available budget | The experts agree that this action has high impact and high *feasibility* because they consider that there is sufficient budget for the financing of the different policies, especially those focused on anaemia and chronic malnutrition in children.  They also indicated that because of the context caused by COVID-19, this budget decreased.  Furthermore, the experts indicated that it is not enough to have a sufficient budget; it is necessary to ensure that it is well executed. | "*Well with the anaemia issue there has been enough budget, the issue is that last year with the COVID-19 pandemic issue, all programmes had their budget cut, including anaemia. So in terms of allocating budget, I would put very high impact and high feasibility for the same reason that there was political support*." *(National representative, government sector)*  "*The expected impact and feasibility is high, because we have already had for many years the Articulated Nutritional Program where budget is allocated mainly for the issue of anaemia and chronic undernutrition and this budget has been increasing, the problem is that in the context of COVID-19 pandemic, the budget of these programs is being allocated to the pandemic*." *(National representative, government sector)*  "*What happens is that the budget is not only that it is sufficient but that it is well executed, especially that because the big problem in the public sector is the execution, the impact would be high and the feasibility would depend on the execution we have to look at the last years if they have really executed it and they have not executed it, then the problem is not in that they give more but in that they execute it well*." *(National representative, government sector)* |
