# Supplementary material for: Identifying priority double-duty actions to tackle the double burden of malnutrition in infants and young children in Peru: Assessment and prioritisation of government actions by national experts
Source: PLoS One. 2024 May 20;19(5):e0303668. doi: 10.1371/journal.pone.0303668 (PMC11104715; doi:10.1371/journal.pone.0303668)
Supplement: Attachment — Submitted filename: Response to Reviewers.docx [file pone.0303668.s009.docx]

**Rebuttal letter**

We would like to thank the two reviewers for the time they spent reviewing our manuscript and for their valuable comments which strengthened our paper. Please find below a detailed response to both reviewers.

| **Points raised by the journal** | **Authors’ responses** |
| --- | --- |
| 1. Please ensure that your manuscript meets PLOS ONE's style requirements, including those for file naming. The PLOS ONE style templates can be found at  <https://journals.plos.org/plosone/s/file?id=wjVg/PLOSOne_formatting_sample_main_body.pdf> and  <https://journals.plos.org/plosone/s/file?id=ba62/PLOSOne_formatting_sample_title_authors_affiliations.pdf> | We have reviewed the style requirements including the file naming requirements and made changes according to the PLOS ONE style templates. |
| 2. In the ethics statement in the Methods, you have specified that verbal consent was obtained. Please provide additional details regarding how this consent was documented and witnessed, and state whether this was approved by the IRB | We have included the details requested in the ethical statement.  Ethical approval for the PERUSANO project was obtained from the Ethics Review Committee of the Instituto de Investigación Nutricional (IIN) Peru (reference 388-2019/CIEI-IIN). As the study was conducted during the period of mandatory confinement due to the COVID-19 pandemic, all participants gave verbal informed consent after receiving written (via email) and verbal (via virtual interview) information about the study. Verbal consent was obtained from the participant via virtual interview and this process was recorded and archived in the study's regulatory folder. The procedures for taking verbal informed consent were included in the research protocol and were evaluated and approved by the Ethics Review Committee of the Instituto de Investigación Nutricional (IIN) Peru (reference 388-2019/CIEI-IIN).  This has been added lines 233 to 243 on page 11 |
| 3. Thank you for stating the following financial disclosure:  Funding: This study was supported by the UK Medical Research Council (MR/S024921/1) and CONCYTEC/FONDECYT Perú (032-2019) through the Newton Fund.  Please state what role the funders took in the study.  If the funders had no role, please state: "The funders had no role in study design, data collection and analysis, decision to publish, or preparation of the manuscript."  If this statement is not correct you must amend it as needed.  Please include this amended Role of Funder statement in your cover letter; we will change the online submission form on your behalf. | We have added the requested statement to the cover letter.  "The funders had no role in study design, data collection and analysis, decision to publish, or preparation of the manuscript." |
| 4. Please include your full ethics statement in the ‘Methods’ section of your manuscript file. In your statement, please include the full name of the IRB or ethics committee who approved or waived your study, as well as whether or not you obtained informed written or verbal consent. If consent was waived for your study, please include this information in your statement as well. | We have included the full ethical statement in the "Methods" section of the manuscript with the required details: full name of the IRB or ethics committee that approved the study, how verbal informed consent was obtained:  Ethical approval for the PERUSAN project was obtained from the Ethics Review Committee of the Instituto de Investigación Nutricional (IIN) Peru (reference 388-2019/CIEI-IIN). As the study was conducted during the period of mandatory confinement due to the COVID-19 pandemic, all participants gave verbal informed consent after receiving written (via email) and verbal (via virtual interview) information about the study. Verbal consent was obtained from the participant via virtual interview and this process was recorded and archived in the study's regulatory folder. The procedures for taking verbal informed consent were included in the research protocol and were evaluated and approved by the Ethics Review Committee of the Instituto de Investigación Nutricional (IIN) Peru (reference 388-2019/CIEI-IIN).  This has been added lines 233 to 243 on page 11 |
| 5. Please remove your figures from within your manuscript file, leaving only the individual TIFF/EPS image files, uploaded separately. These will be automatically included in the reviewers’ PDF. | We have removed the figures from the manuscript file, and uploaded the individual TIFF/EPS image files separately. |

| **Reviewer #1** | **Authors’ responses** |
| --- | --- |
| 1.. This paper addresses an incredibly important issue, and understanding the potential for nutrition policy actions which can address all forms of the double burden of malnutrition is a pressing priority. The paper has a great research question and overall a good research approach to answering the question. | Thank you for your positive feedback. |
| 2. However, I found the presentation of the results of this analysis to just be too complex and confusing to get a cohesive picture of what is going on in Peru. The authors need to find a way to summarise their findings more effectively, perhaps by collapsing categories or summarising sections, or perhaps by using tables or figures to make the results more digestible. This applies particularly in the Discussion section where I hoped to find some more synthesis. | We have reflected on your comment. We have added a summary of the main findings of this study, which are summarised in two parts at the beginning of the Discussion section: the policy measures that Peru is implementing well and those that need to be strengthened. This summary of findings allows the reader to get a coherent picture of what is happening in Peru.  See lines 485-528 on page 22-23 |
| 3. It would also help in interpreting this information to know what the nutrition policy goals and objectives of the Peru government are to understand more the context of all the multiple policies discussed in the paper. | Thanks for the suggestion. In the introduction, the goals and objectives of the Peruvian government's nutrition policy have been added to better understand the context of all the multiple policies discussed in the document.  This has been added lines 79 to 90 on page 4 |
| **4. Introduction** - This section needs to include more information on the Peru context in two ways. o Firstly what are considered drivers of DBM in this setting, some more background on the nutrition situation in Peru could be included. o What actually is the overarching government policy towards nutrition in the country. What are the stated aims? Is the double burden actually a priority? Are policy developments aimed at this double burden? | We have added background information on the situation of the double burden of malnutrition in Peru, as well as: the country's governmental nutrition policy and stated objectives; the existence of policies aimed at addressing the double burden of malnutrition; and the prioritisation of the double burden of malnutrition in national policies.  This has been added lines 61 to 90 on page 3-4 |
| **5. Methods** - I think that this section needs to be restructured or reordered. I was quite confused until around half way through what you had actually done. - For example you introduce your study participants at the start of the methods and say they will be ‘validating the evidence document’ without us knowing what that is. - I think you could be more clear and step by step in describing your study design. | We have adopted your suggestion and have subsequently reordered the section to help simplify the process for readers.  See lines 94 to 243 on page 4-11 |
| **6. Discussion** - Although we have a ‘Summary of findings’ section I don’t find that this section achieves its aim. There is so much complexity in this paper in indicators, domains, levels of implementation, levels of evidence regarding implementation etc. The authors really need to find an effective way to summarise all this information and give a clear picture about how overall the Peru government is going in achieving impact on DBM. In reading the results and discussion it was really not clear what the government was doing well and what they were not doing well, or what they intended to do well but had barriers to implementing. I am not sure how this can be done with so much material produced by the study, but it needs to be attempted. | Thanks for the suggestion, we have improved the wording of the "summary of findings" section to enhance understanding of what the Peruvian government is doing well and what needs to be strengthened to tackle the double burden of malnutrition in children under 2 years of age.  See lines 485-528 on page 22-23 |
| 7.- As I mentioned in the introduction section you need some information on what Peru government policies are for nutrition. You could return to this in the Discussion and reflect on policy achievements against this, as well as against all of the indicators you have used. | Your suggestion has been adopted and information on the Peruvian government's policies on nutrition has been added to the introduction and discussion section.  See lines 79 to 90 on page 4  See lines 497-512 on page 22  See lines 523-528 on page 23 |
| 8.- Also to broaden the impact of the paper an assessment of what the Peru government has done well and what lessons for the international community more broadly can be drawn from this work would be helpful. | Thank you, your suggestion was included in the discussion section  See lines 497-512 on page 22-23 |

| **Reviewer #2** | **Authors’ responses** |
| --- | --- |
| 1. This manuscript reports a review of government actions to solve double burden of malnutrition in infants and young children in Peru. The study is well designed. The paper is well written with detailed information and includes discussion of different viewpoints on double duty actions. However, there are some points that should be addressed. | Thank you for your overall positive feedback |
| 2. The tool created to assess the DDA in this study is a useful innovation. The authors should discuss how other researchers/countries should modify/adjust it to best suit their situation. | Thank you for the suggestion, which is now included in the section on strengths and limitations.  See lines 672-682 on page 28-29 |
| 3. Some indicators especially the specific micronutrient supplementation may not be applicable to other settings/countries. For example, vitamin A deficiency may not be a problem but iodine deficiency is. So these indicators should be relevant to the country-specific problems/issues. - It might also be helpful to include maternal nutrition during lactation as one indicator. | Thank you for the suggestion, which is now included in the section on strengths and limitations.  See lines 678-682 on page 30 |
| 3. - Nutrition-specific interventions has a limit in reducing DBM prevalence. To further reduce it, nutrition-sensitive interventions by non-health ministries will play a more important role. For infrastructure support indicators, a platform for interaction between health and non-health governmental organizations such as agriculture, trade, education will be useful. | We agree with your point of view. Among the infrastructure support indicators, there is the intragovernmental interaction platform indicator, which was evaluated in this study. We have added more detail on this indicator in the results and discussion section.  See lines 274-278 on page 12  See lines 504-509 on page 22-23 |
| 4. Experts point out that there is a serious shortage of health workers, especially in counselling work. The authors should discuss how to deal with this problem. For example, using digital educational resources to reduce the burden on staff and educate caregivers and families should be considered. | Thank you for the suggestion, we agree, we have added a paragraph about this in the discussion section  See lines 662- 666 on page 29 |
