# Supplementary material for: Identifying priority double-duty actions to tackle the double burden of malnutrition in infants and young children in Peru: Assessment and prioritisation of government actions by national experts
Source: PLoS One. 2024 May 20;19(5):e0303668. doi: 10.1371/journal.pone.0303668 (PMC11104715; doi:10.1371/journal.pone.0303668)
Supplement: Attachment — Submitted filename: Response to Reviewers.docx [file pone.0303668.s010.docx]

**Rebuttal letter**

We would like to thank the two reviewers for the time they spent reviewing our manuscript and for their valuable comments which strengthened our paper. Please find below a detailed response reviewers.

| **Reviewer #2** | **Authors’ responses** |
| --- | --- |
| 1. If the authors have adequately addressed your comments raised in a previous round of review and you feel that this manuscript is now acceptable for publication, you may indicate that here to bypass the “Comments to the Author” section, enter your conflict of interest statement in the “Confidential to Editor” section, and submit your "Accept" recommendation.  Reviewer #2: All comments have been addressed | Thank you for your overall positive feedback |
| 2. Is the manuscript technically sound, and do the data support the conclusions?  The manuscript must describe a technically sound piece of scientific research with data that supports the conclusions. Experiments must have been conducted rigorously, with appropriate controls, replication, and sample sizes. The conclusions must be drawn appropriately based on the data presented.  Reviewer #2: Yes | Thank you for your overall positive feedback |
| 3. Has the statistical analysis been performed appropriately and rigorously?  Reviewer #2: Yes | Thank you for your overall positive feedback |
| 4. Have the authors made all data underlying the findings in their manuscript fully available?  The [PLOS Data policy](http://www.plosone.org/static/policies.action#sharing) requires authors to make all data underlying the findings described in their manuscript fully available without restriction, with rare exception (please refer to the Data Availability Statement in the manuscript PDF file). The data should be provided as part of the manuscript or its supporting information, or deposited to a public repository. For example, in addition to summary statistics, the data points behind means, medians and variance measures should be available. If there are restrictions on publicly sharing data—e.g. participant privacy or use of data from a third party—those must be specified.  Reviewer #2: Yes | Thank you for your overall positive feedback |
| 5. Is the manuscript presented in an intelligible fashion and written in standard English?  PLOS ONE does not copyedit accepted manuscripts, so the language in submitted articles must be clear, correct, and unambiguous. Any typographical or grammatical errors should be corrected at revision, so please note any specific errors here.  Reviewer #2: No | Thank you for your comment, we have checked the typographical and grammatical errors. |
| 6. Review Comments to the Author  Please use the space provided to explain your answers to the questions above. You may also include additional comments for the author, including concerns about dual publication, research ethics, or publication ethics. (Please upload your review as an attachment if it exceeds 20,000 characters)  Reviewer #2: The final version of the manuscript should be re-checked for typological errors and English corrections. For example on line 77, it should read ... 39.8% of children aged 6-59 months suffered from anaemia. | Thank you for your comment. We have again reviewed the final version of the manuscript for typological errors and corrections in English. We have also corrected the error noted in line 77 |
